# Characterization of Resistance to Rhabdovirus and Retrovirus Infection in a Human Myeloid Cell Line

**DOI:** 10.1371/journal.pone.0121455

**Published:** 2015-03-26

**Authors:** Guney Boso, Nikunj V. Somia

**Affiliations:** 1 Molecular, Cellular, Developmental Biology and Genetics Graduate Program, University of Minnesota, Minneapolis, Minnesota, United States of America; 2 Dept. of Genetics, Cell Biology and Development, University of Minnesota, Minneapolis, Minnesota, United States of America; German Primate Center, GERMANY

## Abstract

Viruses interact with various permissive and restrictive factors in host cells throughout their replication cycle. Cell lines that are non-permissive to viral infection have been particularly useful in discovering host cell proteins involved in viral life cycles. Here we describe the characterization of a human myeloid leukemia cell line, KG-1, that is resistant to infection by retroviruses and a Rhabdovirus. We show that KG-1 cells are resistant to infection by Vesicular Stomatits Virus as well as VSV Glycoprotein (VSVG) pseudotyped retroviruses due to a defect in binding. Moreover our results indicate that entry by xenotropic retroviral envelope glycoprotein RD114 is impaired in KG-1 cells. Finally we characterize a post- entry block in the early phase of the retroviral life cycle in KG-1 cells that renders the cell line refractory to infection. This cell line will have utility in discovering proteins involved in infection by VSV and HIV-1.

## Introduction

As obligate intracellular pathogens retroviruses are intimately dependent on host cell factors throughout their life cycle. Since viral genomes only encode a limited number of genes, viruses need to utilize various functions of the host cell machinery to complete their replication. In addition to the cellular cofactors that are exploited by viruses several cellular proteins (restriction factors) have been found to act in an inhibitory role to combat viral infection. Various methods have been used to identify cofactors and restriction factors including genome wide RNAi screening (see for examples [1] and [2]), analyses of host proteins that interact with specific viral proteins (i.e. [3] and [4]) and characterization of cells refractory to viral infection [5], [6].

Our laboratory as well as other groups previously isolated mammalian cell lines resistant to infection by retroviruses using loss-of-function genetic screens [7], [8]. Characterization of some of these cell lines led to identification of multiple host factors that are involved in retroviral infection such as Zinc Finger Antiviral Protein and fasciculation elongation protein zeta-1 [9]. Gain-of-function genetic screens have also helped identify viral co-factors. For example one of the HIV-1 co-receptors, CXCR4, was identified by cDNA library complementation of non-permissive cells [10]. Collectively these and similar studies indicate the usefulness of non-permissive cells in understanding viral-host interactions.

Vesicular Stomatitis Virus (VSV) is an enveloped virus with a negative stranded RNA genome and is a member of the family Rhabdoviridae. Despite causing only mild disease in humans, VSV has been extensively studied due to its potential as an oncolytic agent and the use of its envelope glycoprotein (VSVG) to alter tropism of retroviral vectors [11], [12]. The broad tropism of VSV that is mediated by VSVG also makes pseudotyped retroviruses excellent tools for stable gene delivery. The remarkable broad tropism of VSV indicates that the receptor(s) for VSVG mediated entry must be ubiquitously present in widely differing cell types from different species that have been successfully transduced. Prompted by this observation, several studies have suggested that plasma membrane lipids such as phosphatidyl serine or gangliosides can serve as the cellular receptors of VSV [13], [14], [15]. This contention has been challenged with more direct evidence that phosphatidyl serine is not the receptor for VSV even though it may be involved in a later step following receptor mediated endocytosis [16].

Two recent studies have increased our understanding of VSVG mediated binding to cells. One study demonstrated that gp96, a ubiquitous endoplasmic reticulum chaperone, can rescue a VSVG binding deficiency in a mouse B cell line that was generated by chemical mutagenesis. Based on this observation the authors proposed that gp96 is either directly interacting with the VSVG receptor or is required for the synthesis and expression of the functional receptor [17]. These authors later reported that gp96 is required for the cell surface expression of a narrow range of proteins and among these are members of the LDL receptor family [18]. This observations was explored further with the observation that low density lipoprotein (LDL) receptor functions as the major entry receptor for VSV while the other members of the LDL receptor family are used as the alternate receptors [19].

In this study we investigated the resistance to retrovirus and VSV infection of a lympho-myeloid progenitor cell line—KG-1. Here we show that KG-1 cells are impaired for infection by VSV and VSVG pseudotyped retroviruses caused by a lack of binding by VSVG even though functional LDLR family members are expressed on KG-1 cells. We further demonstrate that the feline endogenous virus envelope RD114 pseudotyped retroviruses are impaired in their entry into KG-1 cells. Entry into KG-1 cells can be mediated by the envelope of 10A1 murine leukemia virus but KG-1 cells also exhibit a postentry block to retrovirus infection. Here we characterize the nature of this block to lentiviral vectors.

## Materials and Methods

### Reagents and Cell Culture

293T, Jurkat, KG-1, Cem-A, Molt and Cem-SS cells were obtained from the American Type Culture Collection (ATCC). 293T cells were maintained in Dulbecco’s Modified Eagle Medium (Cellgro) supplemented with 10% Fetal Bovine Serum, FBS (Gemini Bioproducts). Rest of the cells were maintained in Iscove's Modified Dulbecco's Medium (ATCC) supplemented with 20% FBS.

The following reagents were obtained through the AIDS Research and Reference Reagent Program, Division of AIDS, NIAID, NIH; p24 Monoclonal Antibody (183-H12-5C) from Dr. Bruce Chesebro and Kathy Wehrly, pSV-Ψ-MLV-env- from Dr. Nathaniel Landau.

Goat-anti-mouse-horseradish peroxidase and goat-anti-rabbit-horseradish peroxidase (HRP) secondary antibodies and West Femto enhanced chemiluminescent (ECL) HRP substrate were obtained from Thermo Scientific (Rockford, IL). Rabbit polyclonal Antibody against gp96 was obtained from GeneTex (Irvine, CA). Mouse monoclonal antibody against GAPDH was obtained from ABM (Richmond, BC, Canada). VSVG mouse monoclonal antibody was obtained from KeraFAST. DiI-LDL was obtained from Kalen Biochemical (Montgomery Village, MD) VSV-eGFP was a kind gift from Dr. Asit Pattnaik (University of Nebraska-Lincoln). Measles-GFP was a kind gift from Dr. Stephen Russell (Mayo Clinic, Rochester).

### Plasmid Constructs

Plasmids used for pseudotyped Retrovirus Production: For HIV-1: CSII-EGFP; an HIV-1 based vector encoding for EGFP driven by EF-1a promoter. ΔNRF; encodes for gag, pol, rev, tat and vpu of HIV-1. HIG; an NL4-3 based vector described previously was a gift from Louis Mansky [20]. pRK5-Nef was cloned after PCR mediated amplification of the nef coding sequence from HIV-1 NL4-3 (obtained from the NIH AIDS reagent program). It was cloned into EcoRI and HindIII sites into the expression vector pRK5 (Addgene).

For MLV: pCLMFG-EGFP; an MLV based vector encoding GFP driven by CMV promoter. pCMVgp; encodes for gag and pol of MLV driven by CMV promoter. Vectors used for retroviral pseudotyping: pMDg; encodes for vesicular stomatitis virus glycoprotein, pHCMV-RD114; encodes for RD114 glycoprotein pSV-A-MLV; Encodes for amphotropic (4070A) MLV envelope pRK5-10A1; encodes for amphotropic (10A1) MLV envelope.

### Virus Production and Infectivity Assays

HIV-1 and MLV vectors were generated by transient transfection of three plasmids into 293T cells as described previously [21]. For HIV-1 vectors 15μg of CSII EGFP, 10μg of ΔNRF and 5μg of the relevant envelope glycoprotein encoding plasmid were transfected using the method of Chen and Okoyama [22]. Envelope deficient virus was generated by transfection of 15μg of CSII EGFP and 10μg of ΔNRF. 72 hours after transfection virus was collected and filtered through a 0.45 μM membrane. Filtered virus was concentrated by ultracentrifugation (100,000 × g, 2 hours at 4°C). Viral pellet was resuspended in Phosphate buffer saline (PBS) and aliquots were stored at −80°C. Viral titers were determined by infecting 1 × 10^5^ Jurkat cells with 10 fold dilutions of the viral preparation. 72 hours after the infection EGFP expression was quantified by flow cytometry on a Becton-Dickinson FACScalibur. Same procedure was followed for production of MLV vectors using following plasmids;15μg of pCLMFG-GFP, 10μg of pCMVgp and 5μg of the relevant envelope glycoprotein.

### Reverse transcription products qPCR assay

1 × 10^5^ cells were plated into 6 well dishes and infected at an MOI of 1. Cells were kept at 4°C for 1 hour before and after the addition of virus to synchronize the infections. Controls consisted of uninfected cells or cells infected with heat inactivated virus for 36 hours. Cells were harvested and washed with PBS at different time points; 8 hours for first jump products, 48 hours for 2LTR circles and 6 weeks post infection for full product. Cell lysates were prepared by resuspending the cell pellet in lysis buffer (Tris pH 8.0, 25 mM EDTA pH 8.0, 100 mMNaCl, 1% Triton X-100, and 2 mg/ml proteinase K) and incubating at 55°C overnight. Proteinase K was inactivated by treating the lysate at 95° C for 15 minutes. Lysates were used directly for qPCR analysis. Following primers were used for qPCR: for first jump products; U31—GGA TCT ACC ACA CAC AAG GC, U32—GGG TGT AAC AAG CTG GTG TTC. For 2LTR circles: MH535—AAC TAG GGA ACC CAC TGC TTA AG, MH536—TCC ACA GAT CAA GGA TAT CTT GTC. For Full product: LTR9—GCC TCA ATA AAG CTT GCC TTG, AA55—CTG CTA GAG ATT TTC CAC ACT GAC β-actin- ATC ATG TTT GAG ACC TTC AA, 3' β-actin- AGA TGG GCA CAG TGT GGG T. QPCR reactions using SYBR green were performed using an Eppendorf Real Plex master cycler EP and BioRad SYBR SuperMix according to manufacturer's protocol. Cycling conditions used were 95°C for 2 min, followed by 40 cycles of 95°C 30s, 58°C 30s, and 72°C 30s, and a final extension of 5 minutes at 72°C for all PCR products. Cycle threshold value was used to normalize the DNA amounts for the Jurkat cells. Fold difference was calculated using the Delta Ct method. The melt curve as well as analysis of the PCR products by agarose gel electrophoresis confirmed the presence of one product at the expected size (data not shown). DNA input was controlled by qPCR amplification of a fragment of the β-actin gene.

### Viral entry and binding assays and immunoblotting

For VSVG binding and entry assays 1 x 10^6^ KG-1 and Jurkat cells were incubated with HIV-VSVG (MOI = 5) at 4° C for 1 hour. Infection was started by moving the cells into 37° C. Cells were collected at different time points, washed 10 times with cold PBS. For the entry assay cells were treated with 0.05% Trypsin-EDTA (Life Technologies) for 15 minutes at 37° C to remove surface bound virions and then washed 10 times again with cold PBS. Washed cells were lysed with lysis buffer (20 mM Tris-HCl [pH 8.0], 1 mM CaCl_2_, 150 mM NaCl, 1% Triton) and proteins were separated with SDS-PAGE on 12% polyacrylamide gel. Presence of virus and cell proteins were detected with immunoblotting using the antibodies indicated. Same procedure was followed for RD114 mediated HIV-1 binding and entry assays. Cells were collected at 2 hours post infection and either treated with Trypsin-EDTA (for entry) or just washed (for binding).

Flow cytometry based VSVG binding assay was performed by first incubating 1 x 10^6^ KG-1 and Jurkat cells with HIV-VSVG (MOI = 5) at 4° C for 2 hours. Following extensive washes with cold PBS samples were incubated with anti-VSVG antibody for 1 hour at 4° C. Unbound antibody was washed with cold PBS and secondary antibody with conjugated Alexa488 was added to the cells and incubated at 4° C for 1 hour. Following several washes with cold PBS fluorescent cells were detected using Becton-Dickinson FACScalibur. For the detection of gp96 expression, 5 x 10^5^ KG-1 and Jurkat cells were lysed with lysis buffer (20 mM Tris-HCl [pH 8.0], 1 mM CaCl_2_, 150 mM NaCl, 1% Triton), proteins were separated with 12% SDS-PAGE and gp96 was detected with immunoblotting.

### LDL uptake assay

1 x 10^6^ KG-1 and Jurkat cells were plated into 6 well plates in serum free media. 3 μg/mL DiI-LDL was added and the cells were incubated for 4 hours. The cells were then washed 3 times with PBS and treated with 0.05% Trypsin-EDTA at 37° C for 15 minutes to remove surface bound DiI-LDL. After several washes with PBS, fluorescence of cells was scored using Becton-Dickinson FACScalibur.

## Results

### KG-1 cells are resistant to infection by HIV-VSVG and MLV-VSVG

**Fig 1:**
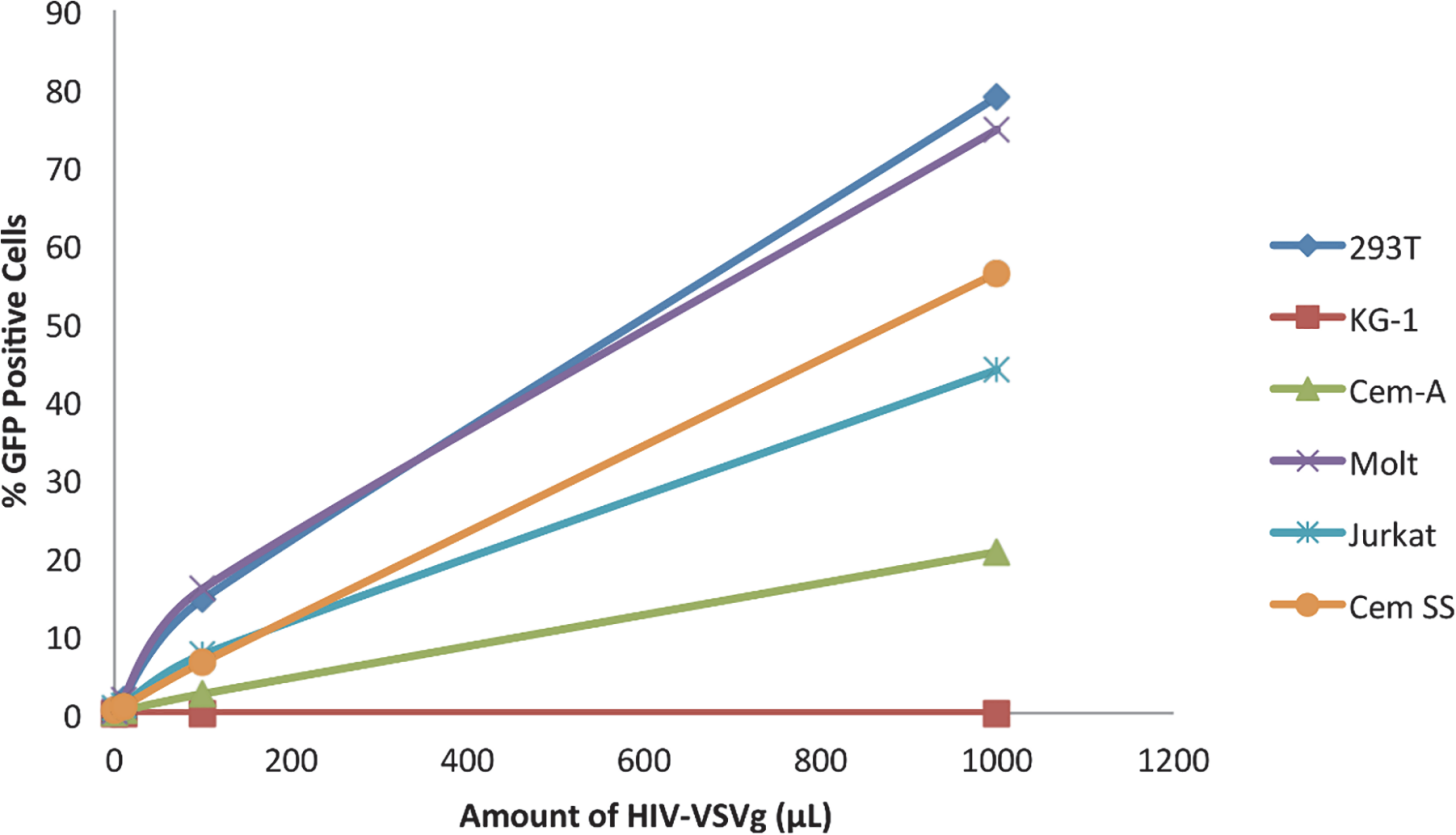
Infectivity of different human cell lines with HIV-VSVG. Several human cell lines were transduced with different amounts (3 log range) of HIV-VSVG that was produced in 293T cells. Percent of EGFP positive cells were determined using fluorescence cytometry 3 days post infection. The Fig. is representative of three independent experiments.

**Fig 2:**
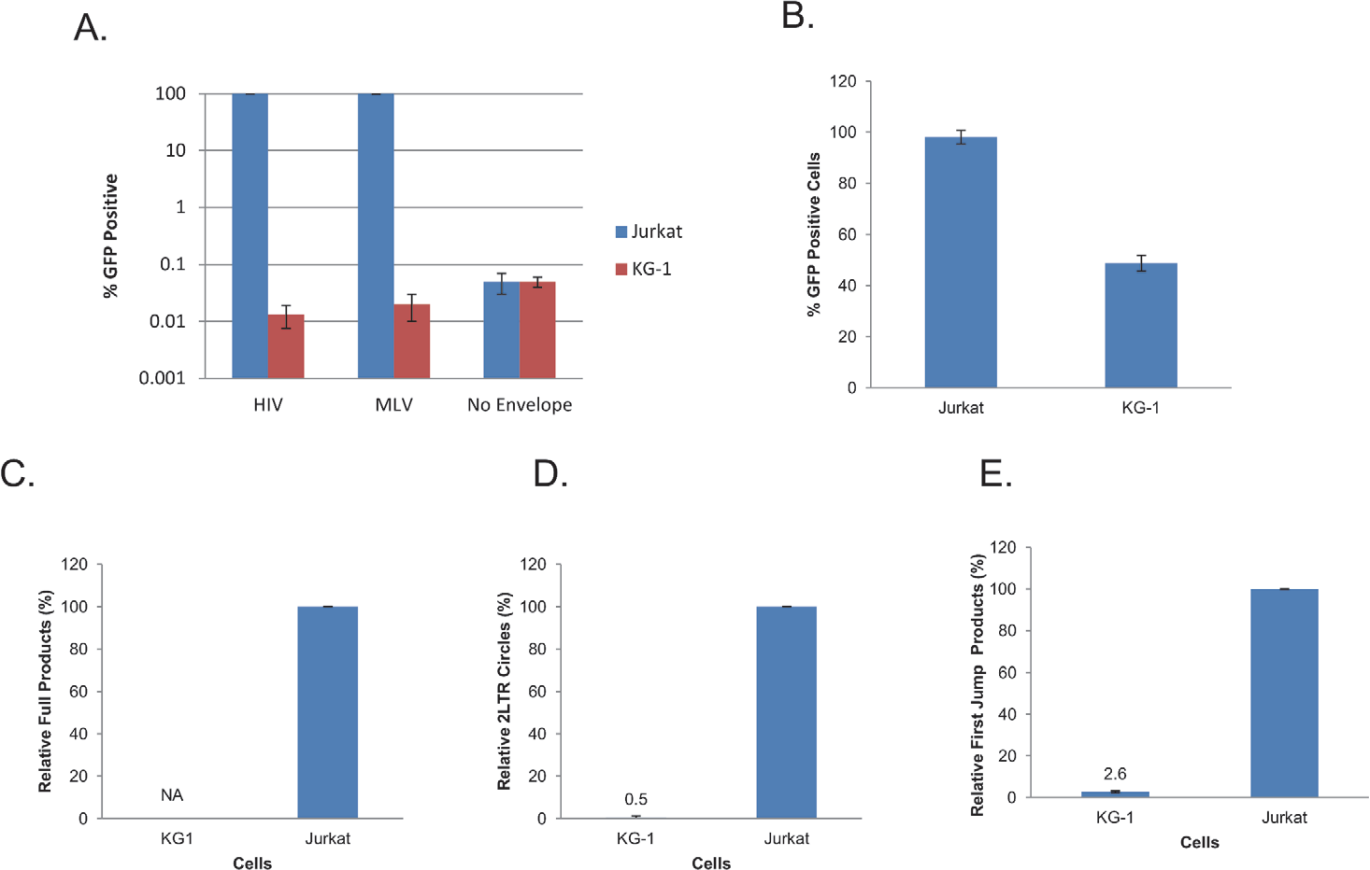
KG-1 cells cannot be infected with VSVG pseudotyped retroviruses. **(A)** KG-1 and Jurkat cells were infected with HIV-VSVG, MLV-VSVG (at an MOI of 10) or with an envelope deficient HIV with p24 levels that were equivalent to HIV-VSVG. Percent of GFP positive cells were determined using flow cytometry 3 days post infection. (**B)** KG-1 and Jurkat cells were infected with Measles virus encoding GFP at an MOI of 3. Percent of GFP positive cells were determined using flow cytometry 48 hours post infection. **(C)** KG-1 and Jurkat cells were infected with HIV-VSVG at an MOI of 1 and viral cDNA products were quantified using qPCR for integrated provirus at 6 weeks post infection; NA—no amplification **(D)** for 2LTR circles at 48 hours post infection and **(E)** for first jump products 8 hours post infection. Error bars are calculated as standard deviations of the mean of at least three independent experiments.

During gene transfer experiments in our laboratory, we observed a differential efficiency in transduction of different cell lines by VSVG pseudotyped HIV-1 (HIV- VSVG) based vectors. Fig. 1 illustrates this for EGFP encoding HIV-VSVG in 6 different human cell lines. While the different cell lines revealed a range of efficiency for the vector we observed no EGFP positive cells in the KG-1 population (Fig. 1). Since non- permissive cell lines can be powerful tools to understand virus-host interactions we decided to characterize the block to HIV-VSVG infection further in KG-1 cells. First we infected KG-1 cells with HIV-VSVG at higher multiplicities to test whether the block to infection can be overcome which can be indicative of a factor that can be saturated. As shown in Fig. 2A, using multiplicity of infection (MOI) of 10 no transduction was observed in KG-1 cells while 100% of Jurkat T-cells were infected. To test whether this block is unique to HIV-1 we used another retroviral vector based on a gammaretrovirus Murine Leukemia Virus (MLV). Again no infection was observed in KG-1 cells incubated with EGFP encoding VSVG pseudotyped MLV (MLV-VSVG) at an MOI of 10 while Jurkat cells were fully infected (Fig. 2A). The 0.01% of gene transduction scored on KG-1 cells was not significant since similar levels are observed on infection using envelope deficient virus particles (using p24 levels that are equivalent to an MOI of 10). These results indicate that KG-1 cells are resistant to VSVG pseudotyped retroviral vectors. The resistance to retroviruses in KG-1 cells raised the possibility of a general block to viral infection in KG-1 cells. To test this possibility we infected these cells with measles virus, a negative stranded RNA virus of the paramyxovirus family. Jurkat and KG-1 cells were infected with measles virus encoding EGFP and infection was scored 48 hours post-infection. As shown in Fig. 2B, KG-1 cells were infected with measles virus approximately 50% less efficiently than Jurkat cells at an MOI of 3. We conclude that the block to retroviral infection we observed in KG-1 cells is not caused by a general resistance to RNA virus infection.

### HIV-VSVG infection is impaired at or before reverse transcription in KG-1 cells

We next determined which stage of the HIV-1 life cycle is impaired in KG-1 cells. We isolated total DNA from Jurkat and KG-1 cells at different time points following infection with HIV-VSVG and tracked the presence of reverse transcription products via quantitative PCR (qPCR) using different primer sets [23]. We further isolated DNA from cells that were cultured for 6 weeks after infection with HIV-VSVG and tested for the presence of integrated provirus in KG-1 and Jurkat cells. As shown in Fig. 2C we detected no HIV-1 proviral DNA in KG- 1 cells infected with HIV-VSVG in contrast to Jurkat cells which is relatively denoted at 100% based on fluorescence analysis. This sensitive molecular analysis confirms our phenotypic observation using the EGFP reporter. Similarly we detected very low levels of 2LTR circles (at least 2 logs lower than Jurkat cells) in KG-1 cells as compared to Jurkat cells at 48 hours post infection (Fig. 2D). While 2LTR circles serve as surrogate markers for defective integration the substantially lower number of molecules detected in KG-1 cells compared to infected Jurkat cells suggest a decrease in viral cDNA that enters the nucleus. In support of this model levels of early reverse transcription (first jump) products 8 hours after infection in KG-1 cells compared to Jurkat cells were also essentially absent (Fig. 2E). Notably the levels of first jump products detected in KG-1 cells were similar to non- infected cells and hence we attribute the signal from KG-1 cells as background (KG-1 first jump control Ct = 7.21 +/- 0.68; KG-1 first jump Ct = 7.63 +/- 0.13). These results indicate the presence of a major block to HIV-VSVG infection at or before reverse transcription.

### KG-1 cells cannot be infected by VSV due to a defect in VSVG binding

**Fig 3:**
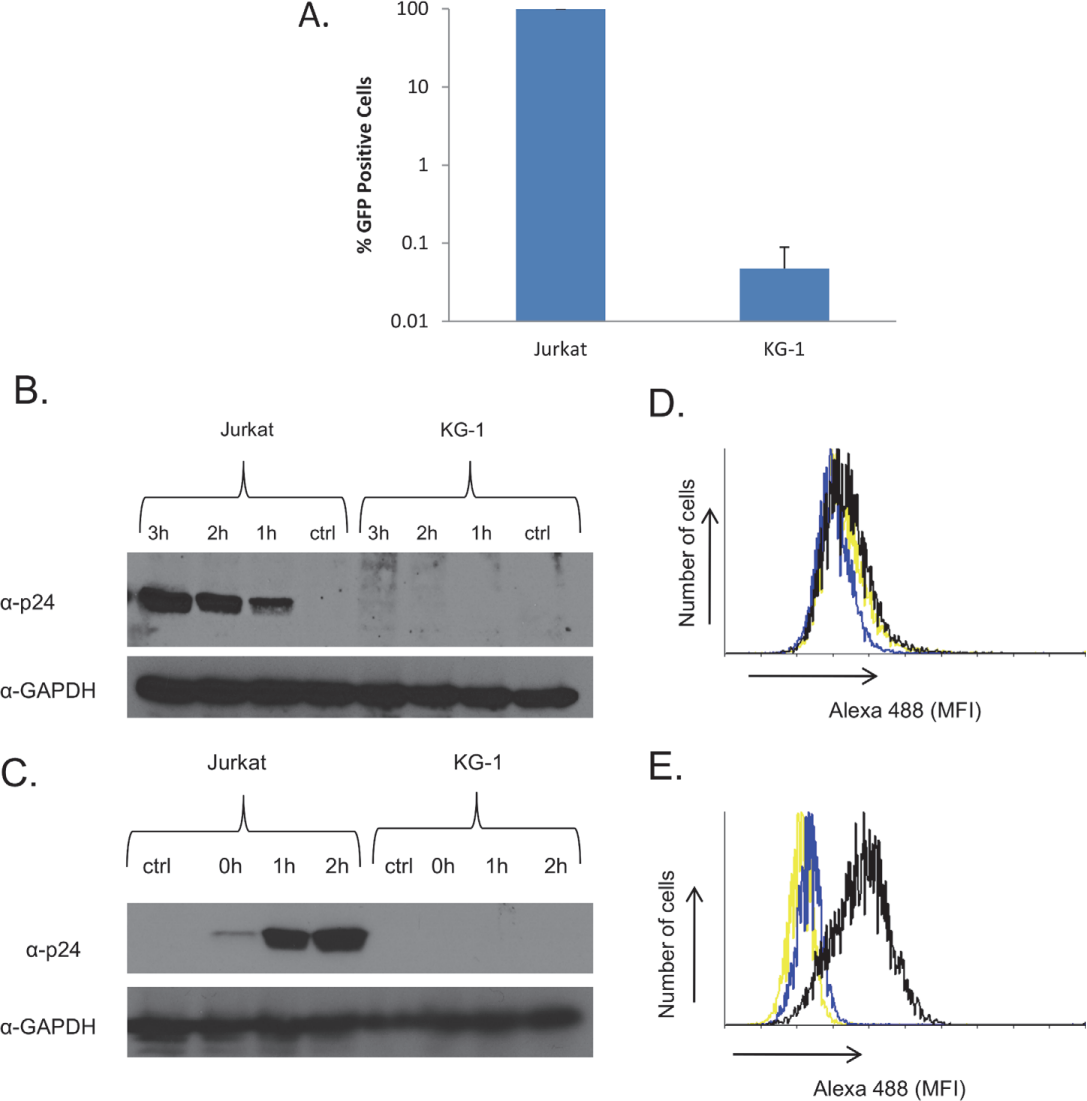
VSVG binding is impaired in KG-1 cells. **(A)** KG-1 and Jurkat cells were infected with VSV-EGFP at an MOI of 50. Percentage of EGFP positive cells were determined at 24 hours post infection using fluorescence cytometry. Error bars are calculated as standard deviations of the mean of at least three independent experiments. (**B)** KG-1 and Jurkat cells were infected with HIV-VSVG at an MOI of 5. Cells were collected at indicated times, washed, treated with Trypsin-EDTA and the lysates were used for immunoblotting with the indicated antibodies. Ctrl indicates non-infected cells. (**C)** KG-1 and Jurkat cells were infected with HIV-VSVG at an MOI of 5. Cells were collected at indicated times, washed and the lysates were used for immunoblotting with the indicated antibodies. The Fig.s are representative of at least three independent experiments. **(D)** KG-1 and **(E)** Jurkat cells were incubated HIV-VSVG at an MOI of 5 at 4°C for 2 hours. Cells were collected, washed and incubated with an antibody against VSVG and an Alexa488 conjugated anti-mouse secondary antibody. Surface bound virions were detected with fluorescence cytometry. Yellow line: Non—infected cells. Blue line: Non—infected cells incubated with antibodies. Black line: Infected cells incubated with antibodies. The histograms are representative of three independent experiments.

To examine the stage before reverse transcription we examined viral entry mediated by VSVG in KG-1 cells. We reasoned that if there was a block to infection because of VSVG, KG-1 cells should also be resistant to infection by VSV. To this end we utilized VSV-eGFP a replication competent VSV that contains the VSV genome with an extragenic eGFP coding sequence between the G and L gene junctions [24]. KG-1 and Jurkat cells were infected with VSV-eGFP at different multiplicities and EGFP expression was tracked by fluorescence cytometry at different time points (data not shown). Fig. 3A shows the result of a VSV-eGFP infection at an MOI of 50 (the titer was determined on baby hamster kidney cells) with EGFP expression measured 24 hours post infection. In contrast to Jurkat cells (100% infection) we observed no EGFP expression in KG-1 cells. These results combined with the results obtained using VSVG pseudotyped retroviruses indicated that there may be a block to VSVG mediated entry in KG-1 cells. To test this possibility further, entry of VSVG pseudotyped retroviruses in KG-1 cells was examined. KG-1 and Jurkat cells were incubated with HIV-VSVG at 4 °C for 1 hour and washed to remove unbound viral particles. Cells were then moved to 37 °C to initiate entry and start the infection. At different time points post infection cells were collected, washed and treated with trypsin to remove surface bound virions. Cells were then lysed and analyzed by immunoblotting for the presence of HIV-1 capsid protein (p24) as an indicator of viral entry. As shown in Fig. 3B, while we detected increasing levels of p24 in Jurkat cells over time no p24 was detected in KG-1 cells. This result suggests an entry defect of HIV-VSVG in KG-1 cells. Next we determined if VSVG binding or internalization is affected in KG-1 cells. Using the same assay we infected KG-1 and Jurkat cells with HIV-VSVG. However the cells were not treated with trypsin and instead washed extensively with cold PBS after collection at different time points post infection. This should allow detection of any bound virus that had not been internalized. As shown in Fig. 3C p24 is detected in Jurkat cell lysates even when cells were kept at 4°C (0 hr time point) where in theory no internalization has occured. In contrast, at the sensitivity for this assay, no p24 was detected associated with KG-1 cells at any point after infection (Fig. 3C). These results indicate a VSVG binding defect in KG-1 cells. We validated this result using a different assay. KG-1 and Jurkat cells were incubated with HIV-VSVG at 4 °C for 1 hour. Cells were then washed extensively with cold PBS and the presence of bound VSVG pseudotyped virus on the cell surface was assayed using a primary antibody against VSVG and secondary anti-mouse antibody containing a fluorescent tag (Alexa 488). Binding was scored using fluorescence cytometry. As shown in Fig. 3D and E while bound VSVG is clearly detected on Jurkat cells (Fig. 3E), no shift in fluorescence and hence no bound VSVG is detected on KG-1 cells (Fig. 3D). Collectively these results indicate that VSVG binding is impaired on KG-1 cells.

### Functional LDL uptake in KG-1 cells

**Fig 4:**
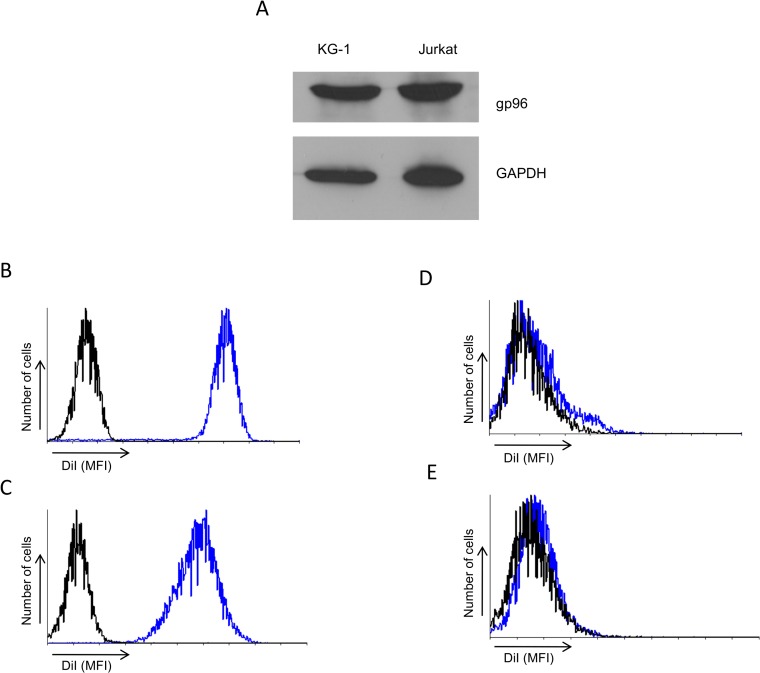
KG-1 cells express gp96 and can functionally take up LDL. **(A)** Immunoblots of KG-1 and Jurkat cell lysates using gp96 and GAPDH antibodies. **(B)** Jurkat and **(C)** KG-1 cells were incubated with DiI-LDL for 4 hours treated with trypsin and fluorescent cells were detected using fluorescence cytometry. Alternatively, cells were treated with trypsin before addition of Dil-LDL to (D) Jurkat or (E) KG-1 cells. (Black line are control cells and blue lines are cells with Dil-LDL added). The histograms are representative of three independent experiments.

A previous report generated a murine cell line through chemical mutagenesis that was found to be impaired in VSVG binding [17]. The results of that study demonstrated that the endoplasmic reticulum chaperone gp96 is required for VSVG binding. To test whether the absence of gp96 can explain the VSVG binding defect in KG-1 cells we probed KG-1 and Jurkat cell lysates for the presence of gp96 via immunoblotting. We observe that gp96 is expressed in KG-1 cells at levels comparable to Jurkat cells (Fig. 4A). Hence a gp96 deficiency cannot account for the defect of VSVG binding to KG-1 cells.

The identity of the receptor for VSVG has remained enigmatic despite the widespread usage of this glycoprotein in gene transfer experiments and the broad tropism of VSV. Results of a recent study suggests that the LDL receptor and its family members can act as the receptor for VSVG mediated entry [19]. Hence we tested whether functional LDL receptor family members are present on KG-1 cells. We utilized an LDL uptake assay using fluorescently labeled LDL (LDL-DiI) [25]. Cells were incubated with LDL-Dil and treated with trypsin and fluorescence was quantified. KG-1 cells showed robust LDL uptake at levels comparable to control Jurkat cells (Fig. 4B and C). We observed the same result when we performed this assay using different concentrations of DiI-LDL and various incubation times (data not shown). Furthermore pretreatment of cells with trypsin before LDL-Dil addition resulted in a lack of binding and uptake (Fig. 4D and E) demonstrating that the trypsin treatment is sufficient to remove the majority of receptor from cells. This result indicates that KG-1 cells express functional LDL receptor family members and that the presence of LDL receptor family members is not sufficient for VSVG mediated binding.

### Entry of RD114 pseudotyped retroviruses is impaired in KG-1 cells

**Fig 5:**
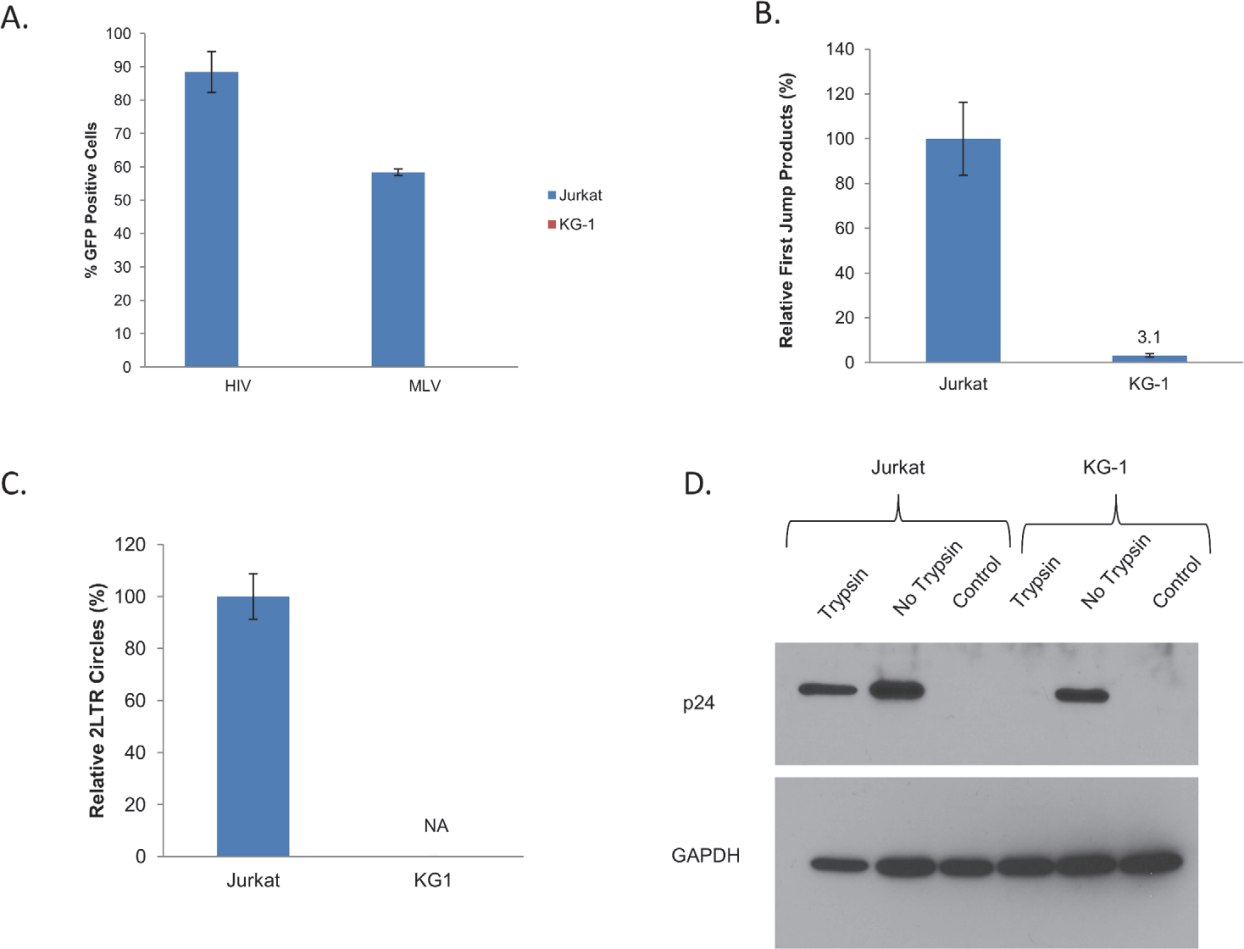
RD114 mediated entry defect in KG-1 cells. **(A)** Jurkat and KG-1 cells were infected with RD114 pseudotyped HIV-1 (MOI = 5) and MLV (MOI = 1) encoding EGFP. Percentage of EGFP positive cells were determined using fluorescence cytometry 3 days post infection. Data are from 3 independent experiments and error bars represent standard deviations of the mean. **(B)** KG-1 and Jurkat cells were infected with RD114 pseudotyped HIV-1 at an MOI of 1 and viral cDNA products were quantified using qPCR for first jump products 8 hours post infection and **(C)** for 2LTR circles at 48 hours post infection. Data are from triplicate samples and are representative of three independent experiments. Errors bars represent standard deviations of the mean; NA—no amplification. (**D)** KG-1 and Jurkat cells were infected with RD114 pseudotyped HIV-1 at an MOI of 5. Cells were collected at 2 hours, washed, treated with Trypsin-EDTA or not and the lysates were used for immunoblotting with the indicated antibodies. Control indicates non-infected cells. The blot is representative of three independent experiments.

Our results above did not allow us to conclude whether there is a block to retroviral infection in KG-1 cells since the resistance we observed for HIV-VSVG and MLV-VSVG seems to be caused by a deficiency in VSVG binding (Fig. 2). To test whether KG-1 cells can be infected by retroviruses we used a different envelope glycoprotein to mediate the entry of our retroviral vectors. To avoid any blocks in viral binding we limited our search to envelope proteins with known receptors that are expressed in KG-1 cells. RD114, the envelope glycoprotein of a feline endogenous retrovirus fits this criteria [26], [27]. The receptor for this glycoprotein has been characterized as a neutral amino acid transporter called SLC1A5 [28]. A search on the GEO profiles database also revealed that this gene is expressed in KG-1 cells (GEO accession GDS2251, [29]). Therefore we produced RD114 pseudotyped EGFP encoding HIV-1 and MLV vectors and tested the infectivity of these vectors in KG-1 and Jurkat cells. As shown in Fig. 5A we observed no infectivity in KG-1 cells using RD114 pseudotyped HIV-1 and MLV while Jurkat cells were infected at high levels with HIV-GFP (85%) and MLV-GFP (55%) at the titers used. We performed qPCR assays to pinpoint the position of this block. Similar to VSVG pseudotyped vectors we observed background levels of early RT (First Jump) products (Fig. 5B), and the absence of 2LTR circles (Fig. 5C) in KG-1 cells infected with RD114 pseudotyped HIV-EGFP. To test whether the RD114 pseudotyped HIV-1 can enter KG-1 cells, KG-1 and Jurkat cells were incubated with the virus at 4 °C for 1 hour and infection was initiated by moving the cells to 37 °C. Two hours post infection cells were collected and either washed extensively with PBS or treated with trypsin to remove particles bound to the cell. Cell lysates were analyzed by immunoblotting for the presence of p24. In contrast to the results obtained with VSVG pseudotyped virus, we detected the presence of virus in the absence of trypsin treatment (Fig. 5D, KG-1, lane labeled no trypsin). However we did not detect any viral capsid protein after trypsin treatment (Fig. 5D, KG-1, lane labeled trypsin).

Receptor for this envelope glycoprotein was discovered via cDNA complementation of NIH3T3 cells which do not support infection with RD114 unless treated with tunicamycin, which inhibits N-linked glycosylation of proteins [28]. Tunicamycin treatment of KG-1 cells did not have any effect on RD114 mediated retroviral entry (data not shown). These results indicate that RD114 pseudotyped HIV-1 vector can bind but cannot enter KG-1 cells.

### KG-1 cells are refractory to retroviruses pseudotyped with amphotropic envelope

**Fig 6:**
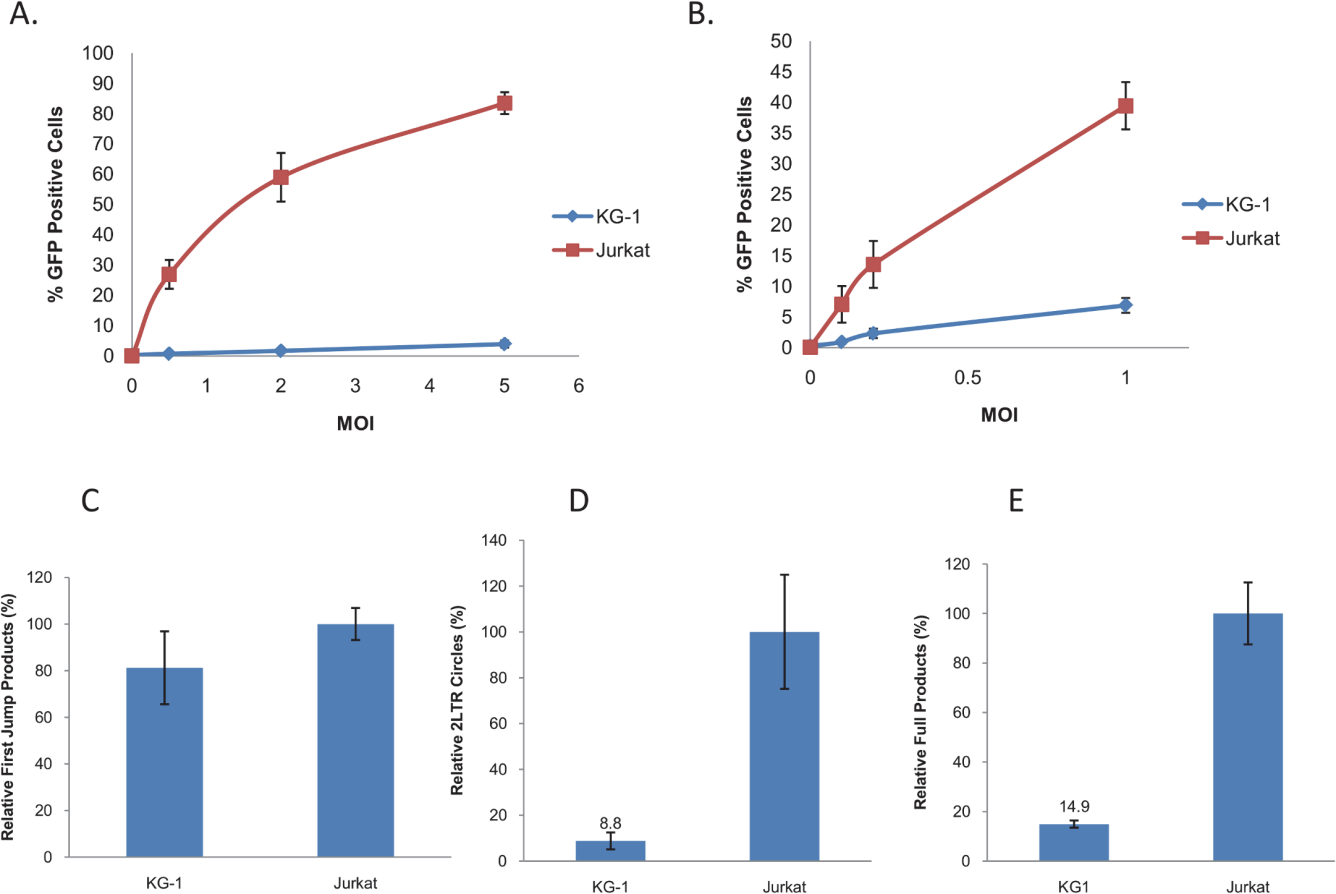
KG-1 cells are refractory to infection with retroviruses pseudotyped with amphotropic envelope. **(A)** KG-1 and Jurkat cells were infected with 4070A pseudotyped MLV-EGFP at different MOIs. Percentage of EGFP positive cells were determined 3 days post infection. (**B)** KG-1 and Jurkat cells were infected with 4070A pseudotyped HIV-GFP at different MOIs. Percentage of EGFP positive cells were determined 3 days post infection. Infection data Data are from 3 independent experiments and error bars represent standard deviations of the mean. **(C)** KG-1 and Jurkat cells were infected with 4070A pseudotyped HIV-1 at an MOI of 1 and viral cDNA products were quantified using qPCR for first jump products 8 hours post infection **(D)** for 2LTR circles at 48 hours post infection and **(E)** for integrated provirus at 6 weeks post infection. Data are from triplicate samples and are representative of three independent experiments. Errors bars represent standard deviations of the mean.

**Fig 7:**
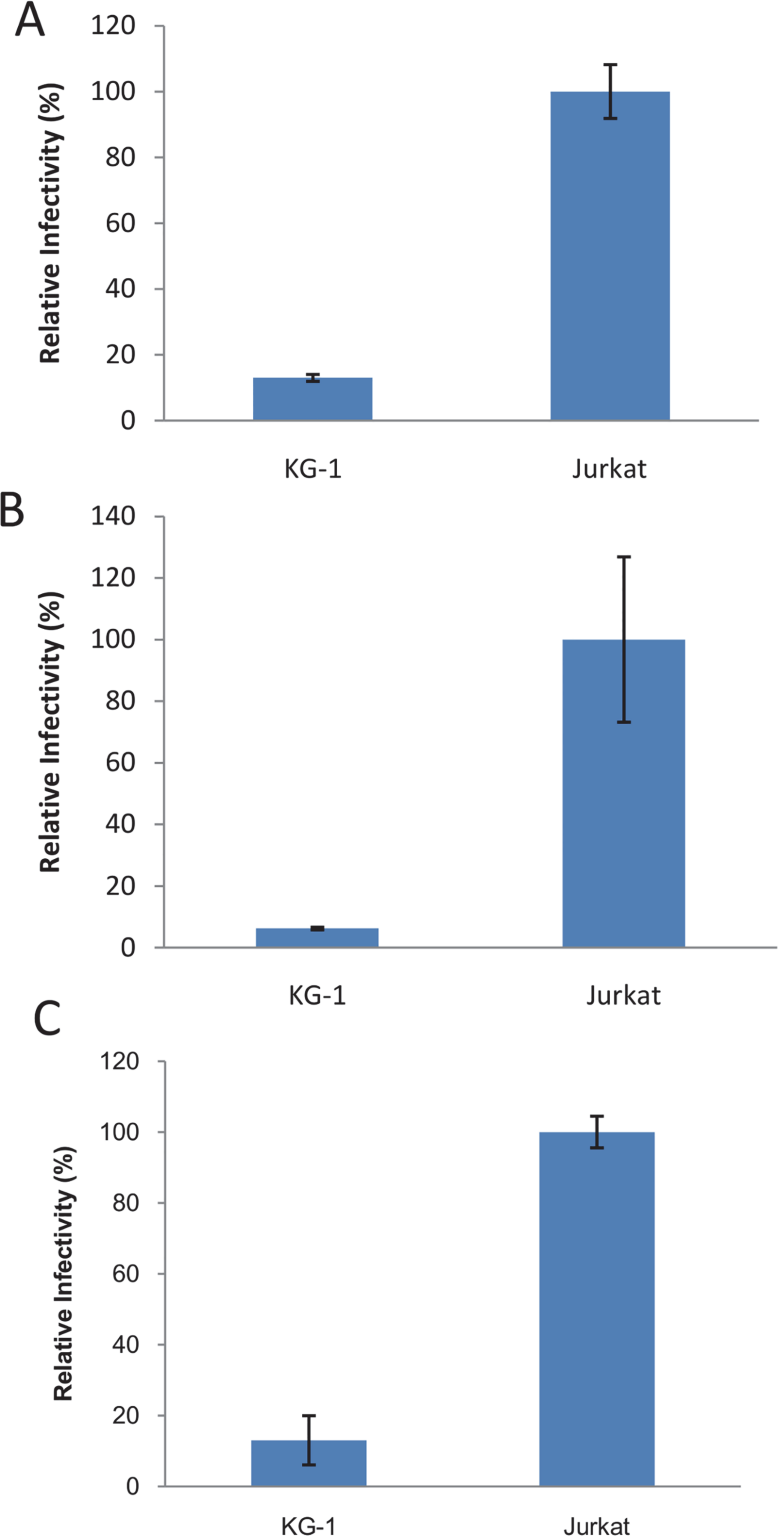
KG-1 cells can maintain integrated provirus and HIV-1 accessory factors doesn't rescue the block to infection. **(A)** KG-1 and Jurkat cells were infected with 4070A pseudotyped HIV-GFP at an MOI of 0.5. Percentage of EGFP positive cells were determined 4 weeks post infection. (**B)** KG-1 and Jurkat cells were infected with 4070A pseudotyped MLV-GFP at an MOI of 1. Percentage of EGFP positive cells were determined 4 weeks post infection. (**C)** KG-1 and Jurkat cells were infected with 4070A pseudotyped HIV-EGFP containing all HIV-1 accessory factors (HIG vector). Percentage of EGFP positive cells were determined 3 days post infection. Data are from 3 independent experiments and error bars represent standard deviations of the mean.

The lack of entry observed for the RD114 pseudotyped retroviruses prompted us to search for another retroviral envelope glycoprotein with a known receptor. We decided to use the amphotropic MLV envelopes 4070A and 10A1 that utilize sodium phosphate co-transporters Pit1 and Pit2 for entry [30]. Moreover the 4070A enveloped MLV has been previously used to study infectivity of MLV in KG-1 cells [31]. As shown in Fig. 6A, KG-1 cells showed low levels of infectivity (between 20–30 fold less) compared to Jurkat cells with 4070A pseudotyped MLV over a range of MOIs. Similar results were obtained with 4070A pseudotyped HIV-1, but the difference in infectivity between Jurkat and KG-1 cells was less pronounced (6–8 fold, Fig. 6B). We obtained similar results using 10A1 envelope pseudotyped viruses and similar results were also observed when a different reporter (Ds-Red) and a different promoter driving reporter expression (CMV) in the vectors were used (data not shown). To better pinpoint the stage in the HIV-1 life cycle that is impaired in KG-1 cells we performed qPCR experiments as described above. Levels of early RT (first jump) products were similar between KG-1 and Jurkat cells at 8 hours post infection (Fig. 6C). This indicates not only similar levels of reverse transcription progression but also similar levels of viral entry in Jurkat and KG-1 cells. In contrast to the early RT products, we observed a relative 8–10 fold difference in the amount of 2LTR circles at 48 hours post infection between KG-1 and Jurkat cells (Fig. 6D). Since 2LTR circles serve as surrogate readout of nuclear import these results suggest a defect in late reverse transcription or nuclear import in KG-1 cells. To test whether the difference observed in the number of EGFP positive cells following 4070A pseudotyped HIV-1 infection in KG-1 and Jurkat cells is reflective of the difference in the amount of integrated provirus, we cultured these cells for 4 weeks after infection. The continued culture of infected cells eliminate any unintegrated products by dilution and degradation. We observed a 6–8 fold difference in the levels of integrated provirus between KG-1 and Jurkat cells (Fig. 6E). These observations are consistent with the results obtained for the functional EGFP assay 3 days post infection (Fig. 6B) and 4 weeks post infection (Fig. 7A) and indicate that the decrease is not due to vector inactivation at the level of transcription. The difference in the number of EGFP positive cells between Jurkat and KG-1 cells after infection with 4070A pseudotyped MLV-GFP is also maintained when the cells were cultured long term (Fig. 7B). Accessory factors encoded by lentiviruses have been shown to be crucial in the infection of certain cell types [32]. The helper plasmid we used in this study does not encode for HIV-1 vpr, nef and vif proteins. To test whether these accessory factors can alleviate the block observed in KG-1 cells we utilized a vector that contains all the accessory factors. As shown in Fig. 7C we observed no difference in the relative infectivity of KG-1 cells as compared to Jurkat cells using this vector when compared to the vector that lacked some of the accessory factors (Fig. 6B). We conclude that the observed resistance to infection in KG-1 cells cannot be alleviated by the presence of HIV- accessory factors.

## Discussion

In this study we characterized the resistance to VSV and retroviral infection in a cell line that was previously shown to be refractory to MLV based vectors [31]. Characterization of non-permissive cells—either generated by mutagenesis or naturally occurring—to retroviral infection has been a powerful tool for discovering host proteins involved in viral life cycles. For example with respect to HIV two of the most studied restriction factors, APOBEC3G and TRIM5α as well as the HIV-1 co-receptor CXCR4 were cloned by initially identifying non-permissive cells [5], [6], [10]. The non- permissive cell line we characterized in this study, KG-1, was isolated from a bone marrow aspirate of a patient with acute myelogenous leukemia [33]. Interestingly KG-1 cells show characteristics of myeloid progenitor cells, and are sometimes described as myeloblasts [34], [35]. These properties of KG-1 cells made them a frequent research tool when studying myeloid cancers and gene transfer into hematopoietic progenitor cells. During such studies we observed resistance to both HIV-VSVG and MLV-VSVG vector transduction even at high multiplicities of infection. These and subsequent experiments demonstrating a resistance to VSV infection initiated experiments that examined binding of viruses to KG-1 cells that were mediated by VSVG. In contrast to Jurkat T-cells these experiments revealed no binding mediated by VSVG to KG-1 cells and this can account for the resistance to infection by VSV and VSVG pseudotyped retroviral vectors. To our knowledge KG-1 cells represent the first human cell line that has been identified that is deficient for VSVG binding and entry. Previously a mouse B cell line had been characterized to be deficient for VSVG binding following mutagenesis with a chemical mutagen ICR191 [17]. The authors of that study identified a frameshift mutation in the gene encoding for the endoplasmic reticulum chaperone gp96. Complementation with a functional gene for gp96 restored VSVG mediated binding and entry by retroviral vectors. That report suggests that a client protein or proteins of gp96 may be receptors for VSVG. As a follow up to this study using plasma membrane profiling it was demonstrated that cell surface expression of some of the LDL receptor family members was decreased in the cells lacking gp96 [18]. Our results indicate that KG-1 cells express gp96 and that it is functional since LDLR expression is also normal in KG-1 cells. Hence we conclude that gp96 deficiency is not the mechanism for the VSVG binding defect in KG-1 cells.

A recent publication identified the LDL receptor and its family members as the cellular receptors of VSVG [19]. This work relies on the observation that soluble LDL receptors can bind VSVG and inhibit plaque formation by VSV and the introduction of the LDL receptor to LDL receptor deficient human fibroblasts increased infection by VSVG but not LCMV envelope pseudotyped retroviruses. However since LDL receptor deficient cells can still be infected by VSV it was hypothesized that other members of the LDL receptor family can be utilized by VSVG for binding and entry. This notion has found support in some recent studies [36], [37] demonstrating a decrease in transduction of various cell lines in the presence of anti-LDLR antibodies or soluble LDLR. We examined fluorescent LDL uptake in KG-1 cells, an assay that tests for the functionality of LDLR and family members. Our results indicate that KG-1 cells are capable of functional LDL uptake. We conclude that LDL binding and uptake and hence functional LDL receptor family members are present on KG-1 cells. Therefore, while LDLR receptors may be necessary for VSVG mediated infection they are not sufficient. We postulate that other factors that are absent or possibly inhibit binding are present in KG-1 cells. Since VSV is being considered as an oncolytic agent KG-1 cells represent an example of how cancers may become resistant to VSV infection and this mechanism of resistance warrants further investigation. Furthermore, due to its broad tropism VSVG is a widely used envelope for pseudotyping gene transfer vectors and understanding its binding partners will also inform these studies.

Following the lack of gene transfer into KG-1 cells using VSVG we next used the envelope glycoprotein of a feline endogenous retrovirus RD114 [26], [27] to facilitate gene transfer. Our results suggest that RD114 mediated entry but not binding is blocked in KG-1 cells. It has been previously observed that RD114 pseudotyped viruses enter the cells via a pH independent path [38], [39]. This suggests that fusion of viral and cellular membrane is blocked on KG-1 cells. Hence KG-1 cells will prove useful in dissecting out the stages required for RD114 entry i.e. other cellular factors are required to mediate fusion at the cell surface.

An inhibition to retroviral infection was previously characterized in KG-1 cells using an MLV based vector pseudotyped with amphotropic 4070A envelope glycoprotein [31]. In that study analysis of viral cDNA by southern blotting in the infected cells revealed the absence of integrated provirus while the presence of viral cDNA was detected using Hirt extraction of low molecular weight DNA molecules in the cells. We observed integrated provirus through EGFP expression in KG-1 cells cultured for up to 6 weeks after transduction with MLV pseudotyped with the same amphotropic envelope. We observed similar results with HIV-1 and further observed the presence of proviral DNA in KG-1 cells via qPCR analysis. The discrepancy between our results and the previous observation are probably due to the difference in sensitivity of the assays—qPCR compared to southern blotting. Moreover our qPCR results indicate that HIV-1 infection in KG-1 cells is impaired at the nuclear import or late reverse transcription step. Taking this difference into account the two studies are in agreement showing a block in KG-1 cells to retroviral infection with a normal accumulation of reverse transcription products but decreased integration. Our study also highlights a decrease in the accumulation of 2LTR circles that suggests a block to nuclear import of the viral DNA.

While some reports agree that KG-1 cells are refractory to infection [40] others have reported transduction of KG-1 cells using agents that aid in the binding of viral particles to the cell surface i.e. peptide nanofibrils or polybrene [41], [42]. The transduction observed may be due to a different route of entry mediated by these agents. These agents may also enhance the pseudo-transduction that is observed as green fluorescence when using EGFP reporters in the short term assays (3 days) that are reported. Finally in some reports the provenance of the KG-1 cells was not reported which can be an issue as KG-1 cells can drift and differentiate over time

The retroviral block in KG-1 is unlikely to be mediated by any of the three major post-entry restriction factors identified—APOBEC3G, Trim5α and SAMHD1. APOBEC3G requires expression in producer cells for its action and it was absent in the 293T cells used in this study. Trim5α is a pattern recognition receptor that acts on the viral core and prevents reverse transcription to proceed [6]. Our results indicate that reverse transcription processing is not blocked in KG-1 cells suggesting that abnormally acting human Trim5α is not the reason for the inhibition of HIV-1 and MLV in these cells. Although the effect of TRIM5 as an innate immune sensor independent of capsid binding cannot be ruled out [43]. Finally, SAMHD1 is a deoxynucleoside triphosphohydrolase which can restrict retroviral infection by depleting the nucleotide pool in non-dividing cells [44], [45], [46] resulting in halted reverse transcription. Our data show that reverse transcription is not impeded in KG-1 cells and further it is unlikely that SAMHD1 can have a substantial effect on infectivity in dividing cells that have high levels of nucleotide pools. Although SAMHD1 action at other points in the HIV-1 lifecycle in KG-1 cells cannot be ruled out.

In conclusion, this study identifies multiple blocks to infection by different viruses and viral vectors in KG-1 cells. Our results indicate that KG-1 cells can be useful tools to identify another component of VSVG binding as well as the mechanism of entry via RD114 glycoprotein. Finally understanding the mechanism to the block to retroviral infection in KG-1 cells may lead to identification of a novel restriction factor or a co-factor that is limiting in KG-1 cells.
